# Comprehensive Morphological and Molecular Insights into Drought Tolerance Variation at Germination Stage in *Brassica napus* Accessions

**DOI:** 10.3390/plants13233296

**Published:** 2024-11-23

**Authors:** Guangyuan Lu, Zhitao Tian, Peiyuan Chen, Zhiling Liang, Xinyu Zeng, Yongguo Zhao, Chunsheng Li, Tao Yan, Qian Hang, Lixi Jiang

**Affiliations:** 1 School of Biology and Food Engineering, Guangdong University of Petrochemical Technology, Maoming 525000, China; luguangyuan@gdupt.edu.cn (G.L.); chenpeiyuan@gdupt.edu.cn (P.C.); liangzhiling@gdupt.edu.cn (Z.L.); zengxinyu@gdupt.edu.cn (X.Z.); zhaoyongguo@gdupt.edu.cn (Y.Z.); 2 College of Plant Science and Technology, Huazhong Agricultural University, Wuhan 430070, China; tianzhi_tao@163.com; 3 College of Life Science and Technology, Hubei Engineering University, Xiaogan 432000, China; xgxh2006@163.com; 4 College of Agriculture, Hunan Agricultural University, Changsha 410128, China; yantao@hau.edu.cn; 5 College of Agriculture and Biotechnology, Zhejiang University, Hangzhou 310058, China; 6 Institute of Crop Science, Zhejiang University, Hangzhou 310058, China

**Keywords:** *Brassica napus* L., seed germination, drought tolerant, GWAS

## Abstract

Drought constitutes a noteworthy abiotic stressor, detrimentally impacting seed germination, plant development, and agricultural yield. In response to the threats imposed by climate change and water paucity, this study examined the morphological divergence and genetic governance of drought resilience traits at the germination stage in 196 rapeseed (*Brassica napus* L.) lines under both normal (0 MPa) and drought-induced stress (−0.8 MPa) scenarios. Our study showed that the composite drought tolerance D value is a reliable index for identifying drought resilience. Through a genome-wide association study (GWAS), we uncovered 37 significant SNP loci and 136 putative genes linked to drought tolerance based on the D value. A key discovery included the gene BnaA01g29390D (*BnNCED3*), encoding 9-cis-epoxycarotenoid dioxygenase, which exhibited significantly heightened expression levels in drought-resistant accessions (*p* < 0.01), underscoring its potential as a positive drought stress regulator and a suitable candidate for genetically enhancing drought resilience. Moreover, we pinpointed four stress-reactive transcription factors (BnaA07g26740D, BnaA07g26870D, BnaA07g26910D, and BnaA07g26980D), two E3 ubiquitin-protein ligases (BnaA05g22900D and BnaC06g28950D), two enzymes (BnaA01g29390D and BnaA03g48550D), and two photosystem-associated proteins (BnaA05g22950D and BnaC06g28840D) as vital components in drought response mechanisms. The construction of a regulatory network reveals an ABA-dependent pathway (*NCED3/RGLG5/IDD14*) that contributes to drought tolerance in rapeseed seedlings, alongside the involvement of a drought avoidance strategy (*APRR6/PHYB*). The SNPs and genes unveiled in this study offer a substantial theoretical foundation for subsequent investigations targeting genetic improvement for drought resilience during seed germination in rapeseed.

## 1. Introduction

Rapeseed (*Brassica napus* L.) is a major oilseed crop, contributing significantly to China’s edible vegetable oil supply. Annually, 7.0 million hectares of rapeseed are cultivated, yielding 15.53 million tons and supplying half of China’s domestically produced vegetable oil (http://www.agri.cn/sc/). Rapeseed also serves as an important bioenergy resource. Its applications include edible oil production, biofuel, livestock feed, and recreational tourism [1]. However, the occurrence of drought poses a serious threat to rapeseed productivity. Drought, as a pervasive abiotic stressor, significantly hinders crop growth, development, and yield, affecting nearly a third of the planet’s arable land [2]. Water scarcity compromises more than half of annual crop yields globally, with seed germination being particularly vulnerable [3].

The development of drought-tolerant cultivars is imperative yet challenging due to the polygenic and multifaceted nature of this trait. Indirect selection criteria, including the Stress Tolerance Index (STI) and Stress Susceptibility Index (SSI), are essential and provide reliable indicators of plant resilience to drought [4]. These indices offer distinct perspectives on drought tolerance mechanisms, enriching our understanding and breeding strategies. Factor analysis emerges as a fundamental analytical tool in the intricate quest to unravel crop drought resilience. By distilling complex datasets from various morphological traits into interpretable factors, it illuminates the complex dynamics governing plant-water interactions, as evidenced in studies on cotton, sweet potato, and barley [5,6,7]. However, the application of such advanced statistical methodologies to decipher drought tolerance in rapeseed remains underexplored, presenting a compelling research gap.

Enhancing crop drought tolerance through genetic means necessitates a comprehensive exploration of the underlying mechanisms operative during the seedling phase, as well as the identification of genetic variation conferring tolerance within species. Genome-wide association studies (GWASs) have proven instrumental in elucidating the genetic architecture of complex traits and identifying candidate genes implicated in desirable phenotypes. Single nucleotide polymorphisms (SNPs), recognized for their abundance and dispersion throughout the genome, have become the preferred markers for GWASs in diverse taxa [8]. This methodology has successfully unraveled drought resistance mechanisms in several crops, including rice [9], barley [10], and wheat [11]. In rapeseed, GWASs have contributed to significant advancements in understanding the genetic control of agricultural traits, such as pod shatter resistance, oil content, seed vigor, yield, and flowering time [8,12,13,14,15]. Notably, Zhang et al. disclosed 16 genomic loci and 79 candidate genes related to water stress responses in canola [16]. Extending this approach, a GWAS conducted on 520 *B. napus* lines illuminated genetic determinants of germination performance under drought [17]. Despite these advancements and the development of drought-tolerant cultivars, the molecular underpinnings of drought tolerance remain only partially decoded, highlighting the necessity for continued investigation to fully harness the potential of genetic resources for crop improvement under water-limited conditions.

Plants have evolved intricate sensing and signaling mechanisms to cope with water scarcity. A critical component of drought sensing is the recently identified receptor DPY1, which acts as an osmosensor, detecting changes in cellular osmotic pressure and initiating signaling cascades that regulate plant responses to drought [18,19]. In parallel, the biosynthesis of abscisic acid (ABA) is crucial for drought response, with the *9-cis-epoxycarotenoid dioxygenase 3* (*NCED3*) gene playing a key role in this process. NCED3 encodes an enzyme that converts carotenoids to xanthoxin, representing the first committed step in ABA biosynthesis [20]. Increased levels of ABA trigger a range of physiological adaptations, including stomatal closure to minimize water loss and the activation of stress-responsive genes that enhance drought tolerance [21]. Additionally, low levels of reactive oxygen species (ROS) act as crucial signaling molecules during drought conditions, influencing the expression of stress-related genes in both ABA-dependent and ABA-independent signaling pathways [22]. This process aids in the mitigation of drought stress and enhances the plant’s adaptive responses.

The primary objective of this research was to investigate the genetic basis of early seedling drought resilience in rapeseed, as drought frequently occurs during autumn, affecting seed germination and emergence in China. We conducted a comprehensive evaluation of 196 rapeseed accessions originating from diverse geographical regions under both normal and simulated water stress conditions using PEG 6000 during germination. Four key traits were assessed, and a composite D value was derived through factor analysis for GWASs. Our primary aim was to identify SNP markers and genes associated with early seedling drought resilience, intending to enhance our understanding of rapeseed drought resistance at the germination stage. This knowledge could potentially contribute to the development of strategies to ensure the security of oilseed crop production, which is crucial for maintaining a stable supply of edible oils.

## 2. Results

### 2.1. Effects of Drought Stress on Seed Germination

We analyzed 196 rapeseed accessions to assess morphological traits during seed germination, including final germination percentage (GP), fresh weight per plant (FW), shoot length (SL), and root length (RL). Descriptive statistics were performed under both normal water supply conditions (0 Mpa) and PEG-simulated drought conditions (−0.8 Mpa), revealing substantial phenotypic variations among the lines (Table S1). There were statistically significant differences between the two treatments for all four traits, with reductions of 72.09%, 71.88%, 83.62%, and 83.86% for GP, FW, SL, and RL, respectively. Under normal water supply, the GP ranged from 92% to 100%, with an average of 97.2%, indicating a high level of seed vigor. However, under drought conditions, the average GP for the 196 lines dropped significantly to 27.1%. This decrease can be attributed to the effects of drought stress during the germination phase (Table S1). The impact of drought stress was also evident in FW, SL, and RL, with the highest coefficients of variation (CV) observed for GP, SL, and RL under drought conditions, reaching 99.4%, 112.2%, and 100.9%, respectively. In contrast, the highest CV for FW was observed under control conditions, at 64.0%. The lowest CV was recorded for GP in the control condition, at 4.3%, reflecting the high initial seed vigor. Furthermore, the rapeseed lines exhibited diverse responses to drought stress, with R4374 and 26 other accessions displaying the lowest GP of 0%, indicating the poorest drought tolerance. Conversely, genotype R4810 achieved a maximum GP of 92%, demonstrating the strongest drought tolerance for this particular line (Table 1 and Table S1). These findings exhibited the varying sensitivity and resilience of rapeseed accessions to drought stress during germination.

To further evaluate drought tolerance performance in the association panel, we calculated the drought tolerance index (DI) for the above four traits (GP, FW, SL, and RL). Based on the four DI indicators, we separately assessed the drought tolerance of the association panel. The results revealed considerable variation in the DI values among the 196 accessions. Specifically, the GP displayed the widest range of DI values, from 0 to 3.25, indicating significant variation in drought tolerance and its suitability as an indicator. In contrast, the FW showed a relatively narrower range of DI values, from 0.017 to 1.373, with less variation. The SL and RL exhibited moderate ranges of DI values, from 0 to 2.77 and 0 to 2.56, respectively, indicating intermediate levels of variation stress (Table S1). These findings underscore the varying sensitivities of different indicators of drought.

### 2.2. Correlation and Factor Analysis

The Pearson’s correlation analysis conducted on the DI values for the four traits revealed significant positive correlations among these indices (Table 2). The most robust correlation was observed between FW and SL, with a high and significant correlation coefficient of *r* = 0.875 **. This indicated a strong interdependence between these two traits. Similarly, GP also exhibited significant positive correlations with FW, SL, and RL (*r* > 0.691 **), suggesting that improvement in GP was likely to be accompanied by concomitant enhancement in FW, SL, and RL.

Although each of the four traits provides valuable information on drought tolerance, their interactions complicate accurate assessment. Therefore, the development of novel composite indicators that integrate these closely associated traits is essential for a more holistic and precise evaluation of drought tolerance. Factor analysis is an effective tool for simplifying the complexity of datasets and identifying key traits that significantly impact drought tolerance. The results of the factor analysis revealed the extraction of four factors, with eigenvalues of 3.315, 0.340, 0.220, and 0.124, respectively (Table 3). Notably, only the eigenvalue of the first factor (F1) exceeded 1.000, indicating its substantial contribution (82.89%) to the overall variance. Consequently, F1 was selected as the primary indicator for a comprehensive evaluation of drought tolerance during germination.

Further characterization of the relative importance of the four component traits (i.e., GP, FW, SL, and RL) for F1 revealed that GP had the highest load of 1.432, while the loads for the other three traits were negative and small (ranging from −0.279 to −0.236). This significant load of GP highlighted its crucial role in assessing drought tolerance during the germination stage. This finding was supported by the high correlation coefficient (*r* = 0.967 **) between F1 and GP, as reported in Table 2. This correlation confirmed that GP could also serve as a robust proxy for evaluating drought tolerance during germination, providing breeders with a valuable tool for screening rapeseed accessions on a large scale.

### 2.3. Comprehensive Evaluation of Drought Tolerance

To assess the drought tolerance of the 196 accessions, a ranking system was implemented. Firstly, the first-factor value (F1) for each accession was normalized using a membership function, resulting in a normalized value known as the DM value. This transformation scaled the minimum factor values to 0, indicating the most drought-sensitive accessions, and set the maximum values to 1, representing the accessions with the highest drought tolerance. Secondly, the DM value for each factor was then aggregated by considering its respective weight, resulting in a comprehensive evaluation value for drought tolerance (D). Thirdly, the levels of drought tolerance for the 196 accessions could be ranked based on these D values. As depicted in Table S2, eight accessions (R5073, R4810, R4474, R4718, R4637, R4796, R4667, and R4905) exhibited the highest D values, ranging from 0.700 to 1.000, indicating extremely strong drought tolerance. This classification was corroborated by their very high GP of 83.67% to 92.00% under drought conditions. Accessions with D values between 0.400 and 0.700, comprising 18 lines, displayed GP varying from 50% to 80% under drought and were considered to possess strong drought tolerance. Accessions with D values ranging from 0.100 to 0.400 (52 lines) exhibited moderate drought tolerance, while those with D values between 0.040 and 0.100 (100 lines) were deemed to have drought susceptibility, with GP ranging from 0 to 42%. Finally, eighteen accessions (R4245, R5032, R4360, R4236, R4374, R4202, R4415, R5067, R4689, R4523, R4743, R4737, R4635, R5044, R4906, R4647, R4748, and R4646) displayed D values below 0.040, indicating the poorest drought tolerance. These accessions had a mean GP of 10.88% under drought conditions, further confirming their high susceptibility to drought.

**Figure 1:**
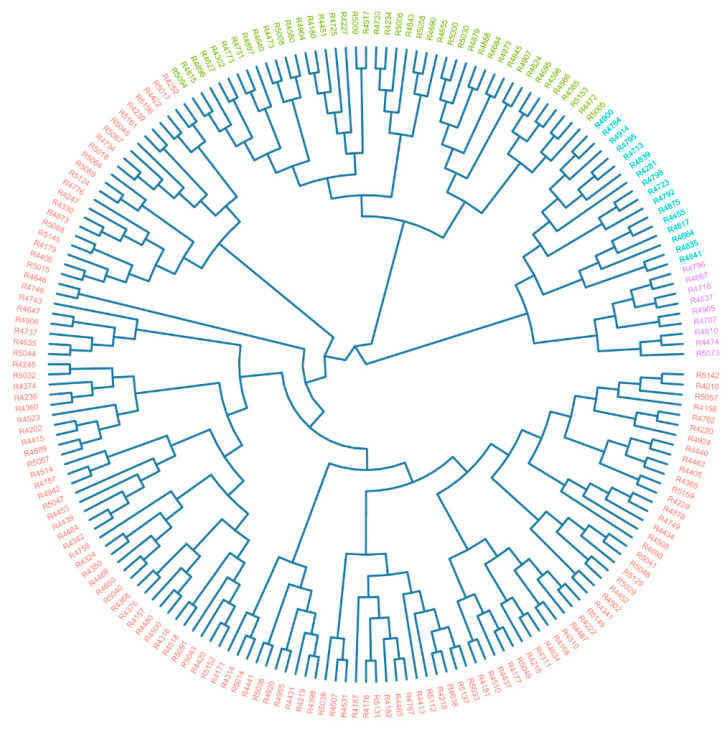
Hierarchical clustering of 196 rapeseed accessions based on D value. All lines were categorized into four groups with different colors.

Cluster analysis has proven to be an invaluable tool in germplasm evaluation, effectively segregating accessions with similar traits into distinct clusters. In our study, the dendrogram generated from the D value successfully grouped the 196 accessions into four distinct clusters (Figure 1). The first cluster (purple) comprised nine accessions with D values ranging from 0.690 to 1.000, indicating exceptional drought tolerance. These accessions primarily belonged to winter types, originating from Germany, Poland, and Japan (Table S3). The second cluster (green) contained 16 accessions, exhibiting strong drought tolerance with D values between 0.420 and 0.610. Most of these accessions originated from European countries and were classified as winter types, with two exceptions being spring types. The third cluster (blue) comprised 42 accessions, displaying moderate to low drought tolerance with D values ranging from 0.140 to 0.390. This cluster was diverse, primarily consisting of winter, semi-winter, and spring types, originating globally. The fourth cluster, represented in pink, encompassed 129 accessions, most of which demonstrated a pronounced susceptibility to drought, as indicated by D values below 0.100. This group also included numerous modern varieties known for their drought-sensitive characteristics. The clustering analysis revealed not only the close relationships among accessions from the same country but also the significant variations in drought tolerance levels across different crop types.

### 2.4. Association Analysis of Drought Tolerance

To pinpoint molecular markers closely linked to drought tolerance during seed germination, a genome-wide association study (GWAS) was carried out with a significance threshold of −log10(*p*) > 5.75, which had been previously utilized in a similar panel [23]. Based on the DI values of four traits, a total of twenty-one association peaks were identified, encompassing five for GP, four for FW, and six for each of SL and RL. These peaks were distributed across chromosomes A01, A03, A04, A05, A07, A09, C03, C04, C06, C07, C08, Ann, and Cnn (Supplementary Figure S1).

**Figure 2:**
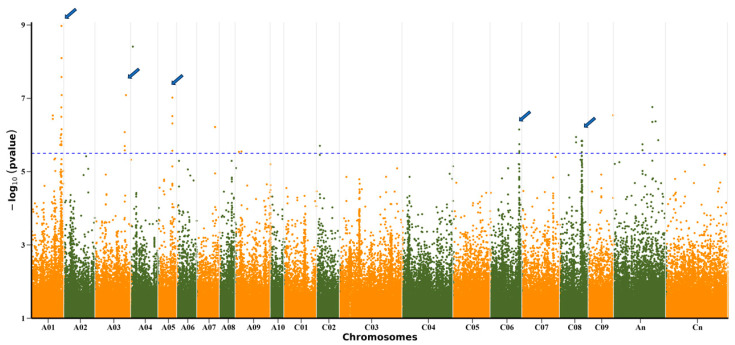
Manhattan plots of GWAS based on D value. Notable association peaks are indicated by arrows. The blue line represents the significance threshold (−log_10_P = 5.75).

Furthermore, the Manhattan plot of the GWAS using the D value revealed 37 significant SNP loci (Figure 2 and Table S4). Notably, over 67% of these significant SNPs were situated on chromosomes A01, A03, C06, and C08, forming four distinct association peaks (Figure 2). Specifically, approximately 40% of the significant SNPs were positioned on chromosome A01, while 12.5% were located on chromosome C08. The remaining significant SNPs were distributed across chromosomes A03, A05, C06, and others (Table S4). Among these, the peaks on chromosomes A01, A03, C06, and C08 coincided with the association peaks detected for the DI of GP (Figure 2 and Figure S1A).

Subsequently, haplotype analysis of the 37 significant SNPs was conducted in 196 accessions (Table 4). Out of the 16 significant SNPs on chromosome A01, 14 were conserved in over 80% of the 196 associations. Notably, the SNPs located at A01_20331510 and A01_20309256 were found to be positioned proximate to the gene BnaA01g29390D at the 5’ and 3’ ends, with physical distances of only 508 bp and 19.9 kb, respectively (Supplementary Tables S4 and S5). Conversely, the SNPs at A07_19610564, A07_19610605, A07_19610615, and A07_19610623 were situated within the gene BnaA07g26830D. This analysis underscores the potential significance of these SNP loci concerning drought tolerance during seed germination.

### 2.5. Candidate Gene Identification and Annotation

To identify potential candidate genes tightly linked to drought tolerance at the germination stage, we conducted a detailed analysis of the 75 kb up- and down-stream regions for each peak SNP identified on chromosomes A01, A03, A05, A07, C06, and C08 through the GWAS using the D value. This analysis revealed a total of 136 candidate genes within the target regions, with 17 genes located on chromosome A01 and 35 genes on A03. Supplementary Table S5 provides the location and annotations of these genes. Remarkably, several of these genes were directly involved in response to drought stress, including BnaA01g29390D, which encodes 9-cis-epoxycarotenoid dioxygenase (*NCED3*), an ortholog of Arabidopsis that plays a crucial role in abscisic acid (ABA) biosynthesis (Table 5). The nucleotide similarity between BnaA01g29390D and *AtNCED3* is 88%. Additionally, we identified four stress-responsive transcription factors (TFs) (BnaA07g26740D, BnaA07g26870D, BnaA07g26910D, and BnaA07g26980D), two E3 ubiquitin-protein ligases (BnaA05g22900D and BnaC06g28950D), two enzymes (BnaA01g29390D and BnaA03g48550D), and two photosystem-related proteins (BnaA05g22950D and BnaC06g28840D) that are associated with drought tolerance during seed germination (Table 5). These candidate genes provided valuable targets for further investigation into the genetic mechanisms underlying drought tolerance.

**Figure 3:**
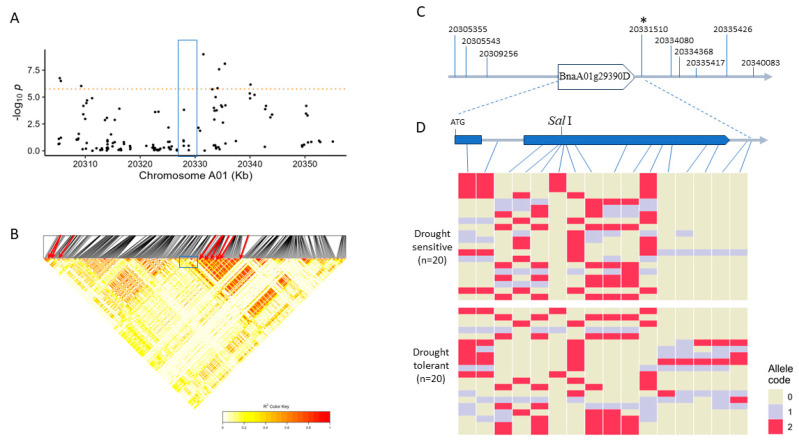
Linkage Disequilibrium (LD) and Haplotype Analysis of Local Region Containing BnaA01g29390D. (**A**), Local Manhattan map of a 50 kb region (from 20.30 to 20.35 Mb) on Chr.A01, highlighting a subset of SNPs shown in the heat map. The position of *BnaA01g29390D* is denoted by the blue rectangle. (**B**), LD heatmap illustrating linkage between nine SNPs indicated by red arrows (corresponding to significant SNPs in (**A**) and the candidate gene BnaA01g29390D (20,329,204 to 20,331,002) in the same 50 kb region, with the approximate position highlighted by a blue rectangle. (**C**), expanded view showing the relative distance between the significant SNPs and BnaC02g16270D. (**D**), Schematic representation of the gene structure of BnaC02g16270D and the top 20 accessions (drought-tolerant) and the 20 accessions with the lowest D value (drought-sensitive). Exons are depicted as solid boxes, and regulatory sequences are indicated by lines. Fourteen SNPs within the gene and two at the 3′-end are shown. The asterisk highlights the significant SNP (A01_20331510). The numbers “0”, “1”, and “2” denote homozygous reference, heterozygous variant, and variant homozygous alleles, respectively. The *Sal*I digestion site at A01_20329724 is shown, which is a target for potential CAPS marker development.

Of particular interest is the candidate gene BnaA01g29390D (*NCED3*), as its crucial role in drought tolerance has been previously documented [24]. To gain further insight into allelic variations for this locus, we conducted a detailed investigation of the 50 kb region surrounding BnaA01g29390D, which included nine significant SNPs. A local Manhattan plot was generated to visualize the positions of these significant SNPs, highlighting the interval on A01 from 20,305,103 to 20,355,103 (Figure 3A). Furthermore, a heat map was created to depict the linkage strength between all significant SNPs, with a focus on the nine significant SNPs and their relationship with BnaA01g29390D, as indicated by color-coded *R*^2^ values (Figure 3B). The heat map revealed a strong linkage disequilibrium (LD) block near the downstream region of BnaA01g29390D. Additionally, a sketch illustrated the positional relationship between the nine significant SNPs and BnaA01g29390D, highlighting their locations within the upstream and downstream regions of the gene (Figure 3C). Notably, the closest SNP linked to BnaA01g29390D, A01-20331510, is only 506 bp away from its 3’ end. Moreover, BnaA01g29390D exhibited five variant homozygous alleles in exon 2 and the 3’ UTR region among the top 20 drought-tolerant rapeseed lines, while the drought-sensitive lines showed only homozygous reference alleles (Figure 3D).

By querying the public rapeseed database BnaSNPDB (https://bnapus-zju.com/bnasnpdb; accessed on 21 February 2024), we identified 15 additional SNPs at the BnaA01g29390D locus (from 20,329,204 to 20,331,520), although below the statistically significant level. These SNPs were distributed across exon 1 (1 SNP), exon 2 (12 SNPs), and the intron (1 SNP) of BnaA01g29390D, as well as the downstream region (1 SNPs) (Figure 3D). Notably, the SNP at A01_20329703 within exon 2 did not result in amino acid alterations. However, a distinct difference in SNP patterns was observed between the top 20 drought-tolerant accessions with high D values and the 20 drought-sensitive accessions with the lowest D values, suggesting a potential phenotypic association of these SNPs (Figure 3D).

Based on the reference genome, specific recognition sites for particular restriction endonucleases were identified within exons 2 of BnaA01g29390D. These include guanine at A01_20329724, thymine at A01_20329736, and guanine at A01_20330225, which can be cleaved by restriction enzyme *Sal*I, *Bcl*I, and *Ssp*I, respectively. *Sal*I, being commercially viable and widely used in laboratories, presents an opportunity for creating a Cleaved Amplified Polymorphic Sequence (CAP) marker to target the recognition sites within the crucial candidate gene, BnaA01g29390D, thus potentially enhancing genotyping and analysis efficiency in future studies.

### 2.6. Expression Analysis of Candidate Genes

To validate the GWAS results, nine candidate genes were selected for expression analysis in drought-sensitive and drought-tolerant accessions under dehydration stress during the germination stage, using quantitative real-time polymerase chain reaction (qRT-PCR). Among these genes, two are involved in hormone synthesis and signaling (BnaA01g29390D and BnaA03g48550D), two function as transcription factors (BnaA03g48570D and BnaA07g26740D), four are associated with photosynthesis and energy metabolism (BnaC06g28840D, BnaA05g22950D, BnaA03g46860D, and BnaA07g26770D), and one is related to environmental stress response (BnaA04g00680D).

**Figure 4:**
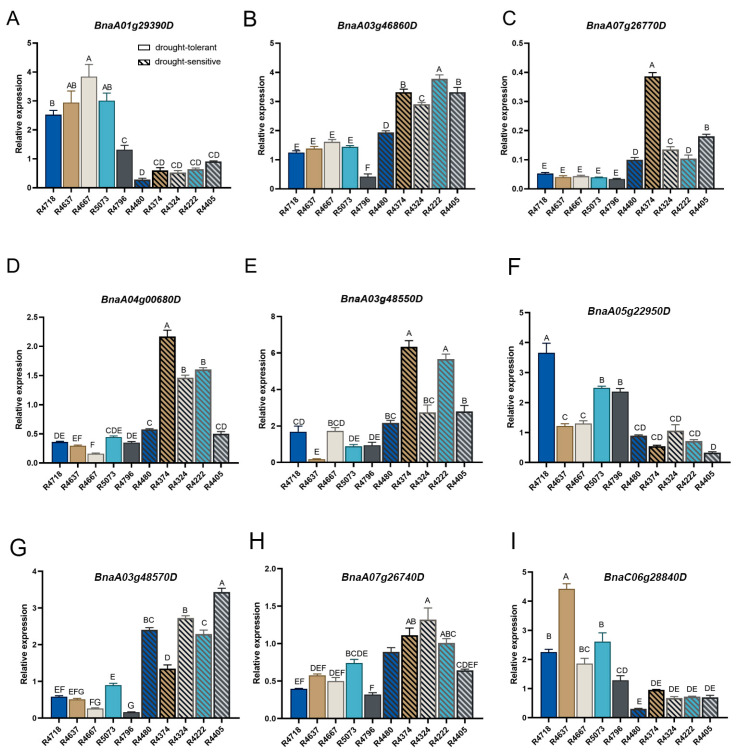
qRT-PCR analysis of the candidate genes. The relative expression levels of nine candidate genes (**A**–**I**) were assessed during the germination stage across ten accessions—comprising five drought-tolerant (R4718, R4637, R4667, R5073, and R4796) and five drought-sensitive (R4480, R4374, R4324, R4222, and R4405) varieties—under both control (0 Mpa) and drought (−0.8 Mpa) conditions. Transcript levels were normalized to *BnACTIN7*, with expression under control conditions assigned a value of 1. The 2^−ΔΔCt^ method was employed for calculation. Error bars represent the standard error (SE) of three biological replicates. Different letters denote significant differences at *p* < 0.01, as determined by one-way ANOVA followed by Duncan’s Multiple Range Test.

The analysis of relative expression levels indicated that BnaA01g29390D (*NCED3*) is a positive regulator of drought stress, as its expression was significantly elevated in drought-tolerant accessions compared to those that are drought-sensitive (*p* < 0.01). A similar expression pattern was observed for BnaA05g22950D (*PHYB*) and BnaC06g28840D (*PSBY*), two genes involved in photosynthesis. In contrast, the expression levels of the other six genes, i.e., BnaA03g48550D (*GH3.5*), BnaA07g26770D (*HPR1*), BnaA03g48570D (*NAC072*), BnaA07g26740D (*HSFA8*), BnaA03g46860D (*ZAT5*), and BnaA04g00680D (*PHA1B*) were notably reduced in drought-tolerant accessions (*p* < 0.01), suggesting a negative regulatory role (Figure 4). Based on the expression data presented above, it can be concluded that BnaA01g29390D (*NCED3*) plays a crucial role in promoting drought tolerance in rapeseed seedlings.

**Figure 5:**
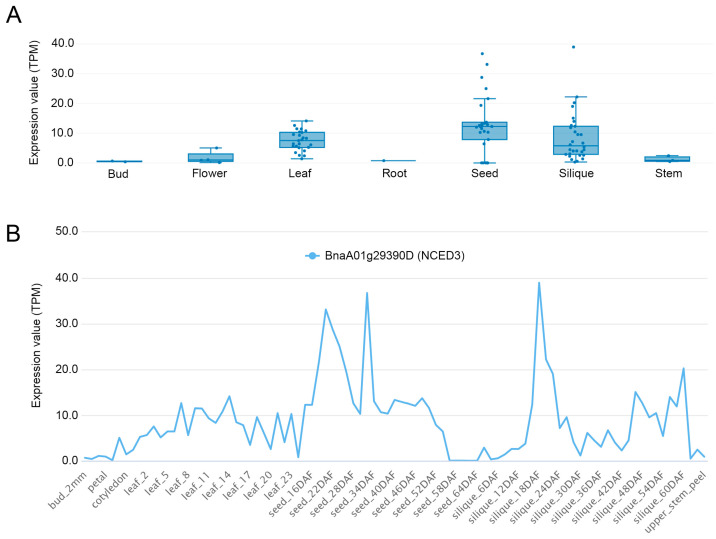
The tissue-specific (**A**) and time-course (**B**) expression patterns of ABA synthetic gene BnaA01g29390D. Expression data were obtained from the public rapeseed database BnIR (Brassica Napus Multi-Omics Database), https://yanglab.hzau.edu.cn/BnIR/expression_zs11 (accessed on 2 November 2024).

We further investigated the spatial and tissue-specific expression profile of BnaA01g29390D using the public rapeseed expression database. The expression of BnaA01g29390D was significantly higher in certain tissues, including seeds, leaves, and siliques, while very low levels were detected in buds and roots, indicating a clear tissue specificity (Figure 5A). Additionally, during seed development, BnaA01g29390D expression steadily increased, reaching its peak at 22 and 34 days after flowering (DAF) before declining to near zero by 64 DAF. In siliques, the highest expression levels were observed at 18 DAF, followed by a decrease characterized by some variability (Figure 5B). This pattern underscores the dynamic regulation of BnaA01g29390D throughout developmental stages and its potential role in seed and silique development.

### 2.7. Interaction Network Between Rapeseed Drought Stress-Related Genes

**Figure 6:**
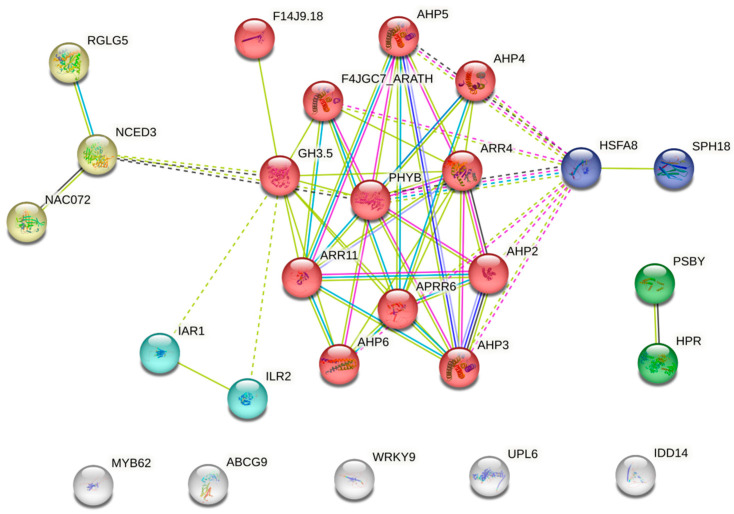
Interaction network of drought-responsive genes identified by GWAS. The group of connected genes was colored differently.

To further elucidate the role of genes in regulating drought response at the protein level, we constructed a protein–protein interaction network utilizing candidate genes identified through the GWAS (Table 5). These genes are involved in hormone signaling, transcription regulation, and photosynthesis. The homologs of the rapeseed candidate genes were identified using the Arabidopsis database and subjected to analysis via the STRING database. The homologs of the rapeseed candidates were categorized into five distinct expression patterns, with interaction lines displaying various associations among the genes. Notably, we identified an ABA-dependent pathway involving three key genes: *NCED3* (BnaA01g29390D) for ABA synthesis, *RGLG5* for ABA signaling (BnaC06g28950D), and *NAC72* (BnaA03g48570D) as a transcriptional regulator. Moreover, *NCED3* interacts with the Auxin signaling pathway *IAR1*/*ILR2* through *GH3*.5 (BnaA03g48550D) and overlaps with the cytokinin-responsive pathway, which consists of 12 genes. Additionally, the heat stress transcription factor *HSFA8* (BnaA07g26740D) exhibited strong interactions with the cytokinin pathway. Furthermore, we detected genes associated with the Photosystem II pathway (*PSBY*), suggesting that both hormonal signaling pathways and photosynthetic networks may play significant roles in the drought response of rapeseed (Figure 6).

### 2.8. Downstream Targets of Drought-Responsive Transcription Factors

**Figure 7:**
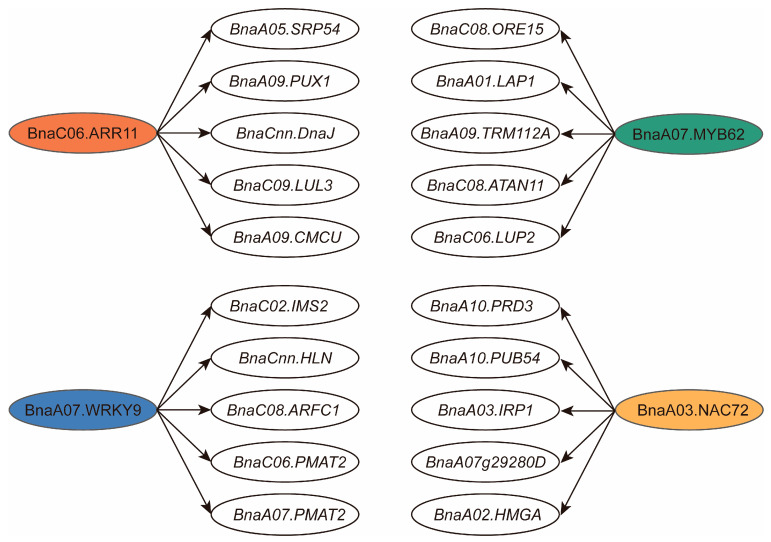
Key downstream target genes are regulated by drought-responsive transcription factors (TFs). Only the top five genes for each TF were listed.

The downstream targets of several drought-responsive transcription factors (TFs) identified through the GWAS were also investigated. For BnaC06g28780D (*ARR11*), a total of 66 putative target genes were identified based on the number of regulatory elements (>4) and their E-values (<1.00 × 10^−30^). The top five target genes include those involved in signal recognition (*SRP54*), plant UBX domain-containing proteins (*PUX1*), chaperone DnaJ-domain superfamily proteins (*DnaJ*), E3 ubiquitin-protein ligases (*LUL3*), and calcium uniporter proteins (*CMCU*). Additionally, BnaA07g26870D (*WRKY9*) primarily regulates methylthioalkylmalate synthase (*IMS2*), ADP-ribosylation (*ARFC1*), and phenolic glucoside malonyltransferase (*PMAT2*). BnaA07g26980D(*MYB62*) is involved in the regulation of leucine aminopeptidase (*LAP1*), methyltransferase (*TRM112A*), and amyrin synthase (*LUP2*). Furthermore, BnaA03g48570D (*NAC72*) regulates genes associated with various metabolic pathways, including Dehydration-Responsive Element-Binding protein (BnaA07g29280D), the ubiquitin-proteasome pathway (*PUB54*), gene expression regulation (*HMGA*), iron homeostasis and metabolic regulation (*IRP1*), and plant’s response to biotic and abiotic stresses (*PRD3*) (Figure 7 and Supplementary Table S6).

## 3. Discussion

### 3.1. The D Value Is a Reliable Indicator for Assessing Drought Resilience in Rapeseed Seedlings

Drought tolerance is a crucial quantitative trait that directly affects plant performance throughout all developmental stages, from seed germination to seed set, as it is intricately linked to the timing and severity of stress across plant development stages [25]. This study assessed the phenotypes of 196 rapeseed accessions under both normal and PEG-mediated drought stress conditions, using various phenotyping indices to evaluate their drought response. Upon subjecting the accessions to simulated drought stress, we observed a significant reduction in GP, FW, SL, and RL (Table S1), highlighting the profound impact of drought on seedling establishment [26]. The substantial diversity in these traits under both control and stress conditions underscores the genetic variability within the association panel. The strong correlation (Table 2) among drought tolerance indices for all four traits (GP, FW, SL, and RL) emphasizes the need for a multi-trait approach for an accurate assessment of drought tolerance, as has been suggested by previous researchers [27].

To enhance population screening and breeding efforts for drought tolerance, we have introduced a good indicator, the D value, which is derived from factor analysis and membership functions. This composite index integrates the DI for traits measured under both stress and control conditions, offering a comprehensive assessment of drought tolerance [28]. Using the D value, we identified a subset of accessions demonstrating outstanding drought tolerance (Supplementary Figure S5), thereby designating them as valuable genetic reservoirs for the development of new crop varieties with enhanced drought tolerance. Furthermore, GWAS analysis utilizing the D value revealed several significant association peaks, highlighting the efficacy of this indicator for phenotyping and genetic elucidation of drought tolerance traits (Figure 2). Our application of the composite indicator, the D value, in genetic studies represents a pioneering effort. Our findings are in line with or even surpass those of Zhang et al., who utilized the root/shoot ratio as an indicator for assessing water stress tolerance in the early seedling growth stages of 140 rapeseed lines [16]. While the root/shoot ratio displayed sensitivity to water stress, showcasing a high degree of variability under stress conditions, only three association peaks were identified based on this indicator under drought conditions [16]. This suggests that the D value may be superior in capturing the complex mechanisms of drought tolerance.

### 3.2. Allelic Variation at BnaA01g29390D Locus Affected Drought Tolerance of Rapeseed

The progress in sequencing technologies and computational biology, paired with a substantial cost reduction, has elevated GWASs to a central role in understanding the genetic architecture of complex quantitative traits in plants, such as drought tolerance [17]. Our rigorous GWAS in rapeseed, utilizing a stringent statistical significance criterion, has unveiled a suite of SNPs closely associated with drought tolerance, a trait critical for enhancing crop performance in arid regions (Supplementary Table S4). The identification of 13 significant association signals on specific chromosomes linked to various drought indices during germination not only unveils genetic diversity but also underscores the complexity of regulating drought responses at the seedling stage. In comparison, Khanzada et al. performed a GWAS at the seedling stage of rapeseed using STI and SSI as measures of drought tolerance, identifying 314 marker-trait associations dispersed across both the A and C genomes [4]. Despite their discovery of 85 drought-related genes, our study, employing a more focused approach and a higher number of SNPs (2,404,340 vs. 201,817), has succeeded in identifying a concentrated set of candidate genes, particularly on chromosome A01. This disparity may arise from our use of a more comprehensive evaluation metric and the higher resolution achieved through a larger SNP dataset.

Of significance, we emphasize a genomic region containing BnaA01g29390D, encoding *NCED3*, a pivotal factor in abscisic acid synthesis, where a cluster of 9 SNPs is located within a 50 kb vicinity, highlighting this locus as a potential center for drought adaptation mechanisms. Indeed, BnaA01g29390D displayed five variant homozygous alleles within exon 2 and the 3’ UTR region in the top 20 drought-tolerant rapeseed lines, whereas the 20 lines characterized as drought-sensitive exhibited homozygous reference alleles (Figure 3). This observation suggests that allelic variation is a critical factor in conferring drought tolerance in rapeseed seedlings. Likewise, Hao et al. discovered that amino acid substitutions in Arabidopsis *AtNCED3* at the 274th site (P→S) and the 327th site (P→R) may play a role in the elevated ABA content observed under drought stress [24]. Furthermore, Liu et al. identified clusters of SNP variations that influence stress-responsive genes in rice [29].

The identified allelic variations hold significant potential for application in marker-assisted breeding. By utilizing the presence of restriction enzyme recognition sites, such as those for *Sal*I, we can potentially develop CAP markers that expedite precise genotyping. This toolkit enables breeders to efficiently introduce drought-tolerant alleles into high-performing cultivars, echoing the successful implementation of similar strategies in soybean breeding, as reported by Zhang et al. [30].

### 3.3. NCED3 Functions as a Key Gene in the Drought Response Regulatory Network

Plants have developed various mechanisms to cope with drought stress, classified into four main strategies: drought avoidance, drought tolerance, drought escape, and drought recovery [31]. Drought tolerance includes delayed senescence, activation of drought-responsive proteins, and hormonal pathways involving ABA, brassinosteroids, and auxins. Drought escape enables plants to complete their life cycle before drought conditions arise, typically through early flowering and grain filling [31]. ABA is crucial for drought adaptation, as it triggers stomatal closure, activates stress-responsive genes, and promotes protective protein synthesis under water-limited conditions. The biosynthesis of abscisic acid (ABA) is primarily facilitated by the *NCED3* gene, which encodes an enzyme that catalyzes the conversion of xanthoxin to abscisic aldehyde, a crucial step in the ABA biosynthetic pathway [20,32,33].

In this study, we identified BnaA01g29390D (*BnNCED3*) through a GWAS (Figure 3). Notably, BnaA01g29390D exhibited significantly higher expression levels, as detected by qRT-PCR, in the seedlings of drought-tolerant genotypes compared to those of drought-sensitive ones (Figure 4). Moreover, allelic variations at this locus significantly influenced drought performance, indicating that BnaA01g29390D plays a crucial regulatory role in drought responses in rapeseed seedlings. Recent studies have also demonstrated that overexpression of *BnNCED3* in both Arabidopsis and rapeseed leads to increased ABA accumulation and enhanced drought tolerance [34,35]. Additionally, transgenic *P. davidiana* plants overexpressing *NCED3* exhibited significantly greater drought tolerance than RNA interference (RNAi) lines that suppressed *NCED* expression [36]. Thus, *NCED3* is an important gene for drought response in plants.

Besides BnaA01g29390D, we also have identified several genes that encode transcription factors (TFs), including BnaA03g48570D, BnaA07g26740D, BnaA07g26870D, BnaA07g26850D, BnaA07g26910D, and BnaA07g26980D, which are homologs of Arabidopsis *NAC72*, *HSFA8*, *WRKY9*, *IDD14*, *APRR6*, and *MYB62*, respectively, that are responsive to drought stress. *NAC72* is a vital member of the NAC domain family of plant-specific transcription factors for the plant’s adaptive response to different environmental stresses, including drought [37]. The *IDD14* gene plays a pivotal role in the plant’s stress response system, including its involvement in abiotic stress responses like drought, as indicated by its interaction with ABFs/AREBs, which are recognized regulators of ABA-dependent stress responses [38]. *APRR6* contributes to the regulation of growth, metabolism, and stress responses within the 24 h cycle as a component of the circadian clock in *Arabidopsis* [39]. Furthermore, genes related to plant hormone metabolism and signaling, including genes encoding *GH3.5* (BnaA03g48550D), *IAR1* (BnaA07g26830D), and *RGLG5* (BnaC06g28950D), were screened out. *GH3.5* is involved in the conjugation of indole-3-acetic acid (IAA), the major plant growth hormone, thus regulating the levels and homeostasis of auxin during growth and development, impacting plant growth and development processes [40,41]. In our study, we found that the relative expression of BnaA03g48550D was significantly reduced in drought-tolerant accessions compared to drought-sensitive ones, indicating that it may function as a negative regulator of drought tolerance. This finding is supported by Casanova-Saez et al., who observed that *gh3.5* mutant plants demonstrated salt-tolerant root growth but were sensitive to PEG-induced water-deficit stress, exhibiting a response similar to that of wild-type Arabidopsis [42]. *IAR1* is involved in auxin signaling, and its overexpression is associated with increased auxin tolerance, indicating a role in regulating auxin sensitivity and response [43].

We also identified genes related to components of the light system, including genes encoding *PSBY* (BnaC06g28840D) and *PHYB* (BnaA05g22950D). PSBY is a crucial component of the Photosystem II complex, essential for light-dependent reactions and adaptation to changes in light intensity during photosynthesis [44]. PHYB, a member of the phytochrome family of light receptors, mediates light-dependent processes such as seed germination, stem elongation, and regulation of flowering time [45]. ARR11 is a type-B response regulator protein crucial in regulating responses to cytokinin, essential for plant cell division and differentiation [46]. HPR1, a peroxidase enzyme, contributes substantially to the structural integrity and function of the plant cell wall. The enzymatic activity of HPR1 has been associated with multiple aspects of plant physiology, including defense responses and the modulation of cell wall characteristics during development [47]. In parallel, our qRT-PCR results suggest that BnaA07g26770D (*HPR1*) functions as a negative regulator of drought stress.

**Figure 8:**
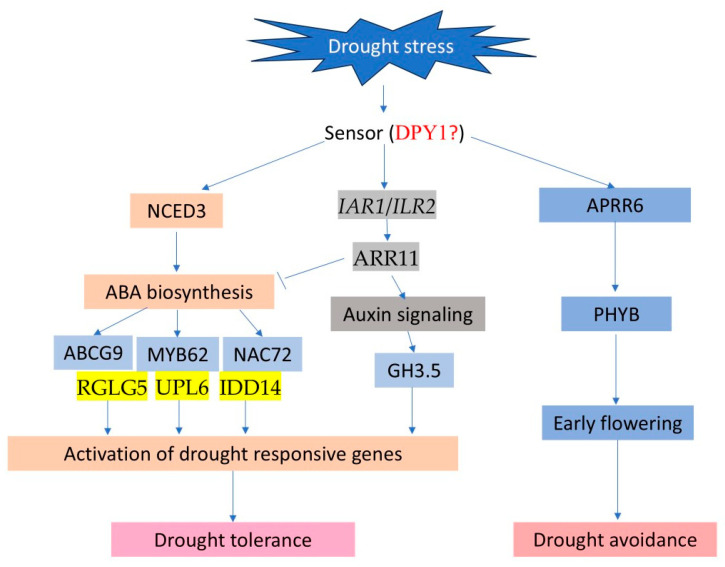
Proposed molecular mechanisms underlying the drought response in rapeseed seedlings.

Based on the genes discussed above, we developed a gene regulatory network and proposed a working model for drought tolerance in rapeseed seedlings (Figure 8). When a water deficit occurs, sensors on the cell membrane first detect osmotic stress, triggering the ABA-dependent pathway. This activation involves the expression of several genes, such as *NCED3*, which synthesizes and accumulates abscisic acid (ABA). The increased ABA levels then activate drought-responsive genes via transcription factors and regulators, including *MYB62*, *NAC72*, *ABCG9*, *UPL6*, *IDD14*, and *RGLG5*, thereby conferring drought tolerance to the plant. Additionally, the auxin signaling pathway is also implicated in the drought response, with crosstalk to ABA-dependent pathways. Lastly, genes related to photosynthesis and the circadian clock, such as *PHYB* and *APRR6*, are also involved, potentially accelerating the life cycle through mechanisms like early flowering to achieve drought avoidance [48,49].

## 4. Materials and Methods

### 4.1. Plant Materials

The 196 rapeseed accessions utilized in this study were maintained at the Crop Research Institute, Zhejiang University, China. The majority of the accessions (n = 136) originated from European countries, followed by China (n = 36). Additional accessions were sourced from Canada, Pakistan, New Zealand, and Australia (Supplementary Table S3). These accessions were categorized into three crop types: winter (n = 113), spring (n = 42), and semi-winter (n = 40). These accessions have previously undergone genotyping and have been successfully employed in the GWASs investigating traits such as tocopherol content, leaf wax, trichome formation, and petal size [23,50,51,52,53]. All plant materials were cultivated at the Agricultural Experiment Field of Hubei Engineering University (113.54° E, 30.56° N) in 2022. Within each row, individual plants were spaced at a distance of 0.15 m, while the distance between rows was maintained at 0.25 m. Seeds were sown at the end of September and harvested in early May of the following year. Field cultivation followed standard local practices. Mature seeds were collected, sun-dried, and stored at -20 °C until further use.

### 4.2. Drought Treatment and Seed Germination

Seeds were germinated in Petri dishes (Bickmann Biotechnology, Changsha, Hunan, China) lined with two layers of filter paper. For each accession, three replicates were employed per treatment, with each biological replicate comprising 50 viable seeds. The filter papers (Whatman, Maidstone, Kent, UK) were moistened with either 7 mL of deionized water (serving as the control) or with polyethylene glycol (PEG) 6000 (Merck KGaA, Darmstadt, Hesse, Germany) solution (as drought treatment) adjusted to an osmotic potential of −0.8 MPa, following the protocols established by Michel and Kaufmann [54]. The Petri dishes were incubated within a growth chamber (SANYO, Moriguchi, Osaka, Japan) maintaining a consistent temperature of 25 °C, a 12 h photoperiod supplied with a light intensity of 100 μmol m^−^^2^ s^−^¹, and a 12 h dark period, all at a relative humidity of 70%. During germination, all Petri dishes were randomly arranged within the chamber and rotated daily to minimize the effects of microenvironmental variation. On day 7 of germination, several parameters were meticulously measured, i.e., GP (germination percentage), FW (the fresh weight of each plant), SL (shoot length), and RL (root length).

### 4.3. Genome-Wide Association Study (GWAS)

A total of 2,404,340 SNPs were extracted from the *Brassica napus* SNP Database BnaSNPDB (https://bnapus-zju.com/bnasnpdb/; accessed on 21 February 2024) and filtered based on minor allele frequency (MAF) greater than 0.05 and missing genotyping rates less than 0.5 to ensure high-quality data. Population structure analysis categorized the accessions into five distinct subpopulations (*k* = 5). To visualize the pattern of LD (linkage disequilibrium) and the distribution of genotypes across the population, heat maps and genotype maps were generated utilizing the dedicated analytical tools provided by the BnaSNPDB platform as described by Yan et al. [55]. For GWASs, the Efficient Mixed-Model Association eXpedited (EMMAX) approach [56] was employed through the BnaVGD platform (https://bnapus-zju.com/gwas/; accessed on 22 February 2024), which Yan et al. [57] have validated for such studies [56,57]. This model accounts for population structure and relatedness, thereby enhancing the accuracy of association tests.

The BnaVGD platform further facilitated the creation of visually informative Manhattan plots and quantile-quantile (Q-Q) plots, which graphically depict the statistical significance of associations and assess the deviation from expected distributions, respectively. Significance thresholds for the GWAS were established at a genome-wide level using a conservative −log10 (*p*-value) of 5.75, consistent with precedent studies by Huang et al. [23], to minimize false-positive associations while capturing biologically meaningful signals.

### 4.4. Candidate Gene Identification, Annotation, and Interaction Analysis

To identify potentially functional genes linked to the detected significant GWAS peak SNPs, a 150-kilobase (kb) region encompassing 75 kb upstream and 75 kb downstream of each peak SNP was scrutinized. This broader genomic window allows a more comprehensive search for candidate genes that could be influencing the trait of interest. Subsequently, the amino acid sequences of the proteins encoded by these candidate genes underwent thorough annotation through homology searches against the Non-Redundant Protein Sequence Database (NRDB) accessible via FTP at ftp://ftp.ncbi.nlm.nih.gov/blast/db/FASTA/nr.gz (accessed on 25 March 2024). This bioinformatics analysis enabled the assignment of putative functions to the candidate genes by aligning them with known protein sequences, thereby providing insights into their potential biological roles and functional classifications.

Protein–protein interactions (PPIs) were analyzed using the STRING v12 database (http://string-db.org; accessed on 02 October 2024) to explore the relationships and functional associations among the putative proteins encoded by drought-responsive candidate genes. The STRING database’s integration of various evidence types provided valuable insights into how these proteins may collaboratively function and interact to enhance the plant’s adaptive responses to drought stress.

### 4.5. Molecular Marker Selection

Based on the distinct phenotypic classifications tied to variations in SNP alleles, the corresponding genotypes for those phenotypes were systematically outlined. To further investigate the genetic factors at play, the DNA sequences of genes suspected to be involved in the observed phenotypes were obtained from the Brassica napus genomic database maintained by Genoscope (http://www.genoscope.cns.fr/brassicanapus/; accessed on 03 May 2024). Utilizing the bioinformatics tool SnapGene (https://www.snapgene.com/; accessed on 06 May 2024), a rigorous screening was conducted to identify those SNPs that give rise to modifications in restriction enzyme digestion sites, a key step in understanding how these genetic alterations could impact gene function and expression.

### 4.6. qRT-PCR Analysis

To validate the expression patterns of candidate genes, ten accessions (five drought-tolerant and five drought-sensitive) were chosen for a germination experiment under both control and drought conditions, following the procedures outlined above. Total RNA was isolated from the germinated seeds using the RNeasy Plant Mini Kit (QIAGEN, Redwood City, CA, USA). Subsequently, the quality and concentration of the extracted RNA were evaluated using an Agilent 2100 Bioanalyzer system (Agilent Technologies, Santa Clara, CA, USA). The synthesis of first-strand cDNA was performed using the EasyScript First-strand cDNA Synthesis SuperMix kit (TransGen Biotech, Beijing, China). Gene-specific primers were designed with precision using Primer3 online software version 4.0.0 (Supplementary Table S7).

qRT-PCR assays were meticulously conducted in 20 μL reaction volumes, consisting of 10 μL TransStart^®^ Tip Green qPCR Supermix (TransGen Biotech), 10 picomoles of gene-specific primers, and 1 μL of synthesized cDNA. These reactions were carried out in an IQ5 thermocycler (Bio-Rad, Hercules, CA, USA), including three technical replicates for each sample. The amplification protocol involved an initial denaturation at 95 °C for 5 min, followed by 40 cycles of 95 °C for 15 s and 60 °C for 30 s. Before using the 2^−ΔΔCt^ method to ascertain relative gene expression levels [58], we performed a normalization of the qRT-PCR data against the endogenous control gene, *actin7*.

### 4.7. Data Processing and Statistical Analysis

The germination datasets from 196 lines under both control and drought conditions were utilized to compute various indices employing Microsoft Excel 2010 [28,59]. The values from the three biological replicates per treatment for each accession were averaged and utilized for subsequent index calculations and GWASs. For each of the four characteristics (i.e., GP, FW, SL, and RL), DC (Drought Tolerance Coefficients) and DI (Drought Tolerance Indices) were individually derived for every accession through the equations:

(1) $${DC}_{i}=\frac{X_{i}}{{CK}_{i}}$$

(2) $${DI}_{i}={DC}_{i}\times \frac{X_{i}}{X_{ai}}$$

where X_i_ symbolizes the attribute’s measurement under drought stress, CK_i_ is the corresponding measurement under control conditions, and X_ai_ denotes the average value of the measurement under drought. Subsequently, factor analysis was employed on the DI to obtain component matrices and summaries explicating the total variance. Notable factors were isolated and harnessed for calculating the DM (Drought Tolerance Membership Values):

(3) $${DM}_{i}=\frac{{DI}_{i}-{DI}_{min}}{{DI}_{max}-{DI}_{min}}$$

with DI_min_ and DI_max_ signifying the minimum and maximum DI values, respectively, for a given trait. The factor weight coefficients ω_i_ were computed as follows:

(4) $$\omega_{i}=P_{i}÷\sum_{i=1}^{n}P_{i}$$

where P_i_ embodies the contribution ratio of the i^th^ composite trait, indicative of its relative significance amidst all traits. Thereafter, integrating the factor weights (ω_i_) and DM, a comprehensive drought tolerance evaluation value, D, was calculated for appraisal of the 196 lines’ drought tolerance:

(5) $$D=\sum_{i=1}^{n}\left[{DM}_{i}\times \omega_{i}\right]$$

Factor analysis, analyses of variance, cluster analysis employing Euclidean distance and the weighted pair group method with arithmetic mean (WPGMA), and computation of correlation coefficients were carried out using IBM SPSS Statistics Version 25.0 (IBM Corporation, Armonk, NY, USA).

## 5. Conclusions

Drought stress during the sowing season consistently negatively impacts the germination of seeds and the emergence of seedlings in rapeseed. To address this challenge, we conducted a comprehensive analysis of the genetic basis of drought resilience in rapeseed seedlings. Using a GWAS panel, we mapped loci linked to four germination-related traits and a derivative composite drought tolerance D value. Within the 150 kb regions surrounding the 37 significant SNPs detected, 136 candidate genes were identified. Among these genes, the one encoding the ABA biosynthesis enzyme, BnaA01g29390D (*BnNCED3*), was subjected to allelic variation and expression analysis. The results showed that this gene was highly expressed only in the drought-tolerant lines and that allelic variation affected drought performance. Furthermore, the constructed regulatory network revealed that the *BnNCED3*-mediated ABA-dependent pathway plays a crucial role in enhancing drought tolerance. Additionally, the *PHYB/APRR6*-mediated photosynthesis and circadian pathway may help rapeseed plants avoid drought by accelerating their life cycle. The SNPs and associated genes identified in this study hold considerable promise for future marker-assisted breeding programs aimed at enhancing drought resistance in rapeseed.

## Figures and Tables

**Table 1:** Descriptive statistics for seedling traits for the rapeseed association panel.

Trait	Treatment	Min	Max	Mean	Coefficient of Variation (%)	*t*-test
Germination percentage (%)	CK	92.0	100.0	97.2	4.3	37.13 ***
	Drought	0	92.0	27.1	99.4	
Fresh Weight (mg)	CK	12.7	223.2	29.3	64.0	16.25 ***
	Drought	2.6	22.8	8.24	43.9	
Shoot length (mm)	CK	5.3	25.3	10.9	36.3	27.58 ***
	Drought	0	7.5	1.8	112.2	
Root length (mm)	CK	16.1	60.5	33.3	24.2	42.76 ***
	Drought	0	23.8	5.4	100.9	

Note: *** indicates statistical significance at *p* < 0.001. CK means normal water supply (0 Mpa), while drought means water deficit (−0.8 Mpa).

**Table 2:** Pearson correlation analysis of the first factor (F1) and drought tolerance index (DI) for four traits.

	GP	FW	SL	RL
Germination percentage (GP)	1			
Fresh weight (FW)	0.733 **			
Shoot length (SL)	0.718 **	0.875 **		
Root length (RL)	0.691 **	0.798 **	0.807 **	
F1	0.967 **	0.564 **	0.533 **	0.510 **

Note: ** means statistical difference at *p* < 0.01 level.

**Table 3:** Factor analysis of drought tolerance index (DI) for four traits during germination.

Trait DI	Factor Load			
	F1	F2	F3	F4
GP	1.432	−0.295	−0.298	−0.380
FW	−0.279	−0.370	−0.767	2.066
SL	−0.236	−0.418	2.061	−0.779
RL	−0.254	1.669	−0.522	−0.445
Eigenvalue	3.315	0.340	0.220	0.124
Contribution (%)	82.885	8.509	5.507	3.099
Cumulative contribution (%)	82.885	91.393	96.901	100

Note: GP, germination percentage; FW, fresh weight per plant; SL, shoot length; RL, root length.

**Table 4:** Allelic variations at significant SNP sites in the association panel.

Location	−LOG10P	Ref. SNP ^a^	%	Variant SNP ^b^	%
A1_16096539	6.53	C	82.5	T	17.5
A1_16096551	6.53	C	82.5	T	17.5
A1_16096597	6.44	G	85.0	T	15.0
A1_20130211	5.92	T	80.0	C	20.0
A1_20237695	6.00	A	62.5	G	37.5
A1_20303192	5.75	G	77.5	A	22.5
A1_20305355	6.75	T	82.5	A	17.5
A1_20305543	6.50	A	80.0	T	20.0
A1_20309256	6.02	G	85.0	A	15.0
A1_20331510	8.98	A	80.0	G	20.0
A1_20334080	5.82	C	82.5	T	17.5
A1_20334368	7.58	T	87.5	G	12.5
A1_20335417	8.10	G	80.0	A	20.0
A1_20335426	8.10	T	80.0	A	20.0
A1_20340083	6.16	T	80.0	C	20.0
A1_20370981	7.09	A	80.0	T	20.0
A3_24029206	6.08	A	35.0	T	65.0
A3_24908344	7.09	A	77.5	G	22.5
A4_518062	8.41	G	77.5	T	22.5
A5_17365548	6.51	A	87.5	C	12.5
A5_17375507	7.02	G	85.0	A	15.0
A5_17375797	6.31	T	87.5	C	12.5
A7_19610564	6.22	T	82.5	G	17.5
A7_19610605	6.22	G	82.5	A	17.5
A7_19610615	6.22	T	82.5	G	17.5
A7_19610623	6.22	C	67.5	T	32.5
C6_30046203	6.15	G	67.5	A	32.5
C6_30085017	5.75	G	72.5	A	27.5
C8_24710445	5.94	A	67.5	G	32.5
C8_24710530	5.80	T	47.5	C	52.5
C8_31254406	5.84	G	47.5	A	52.5
C8_31296364	5.84	T	45.0	C	55.0
C8_31310891	5.83	T	70.0	A	30.0
C9_45802519	6.54	T	92.5	C	7.5
An_33246615	6.36	C	87.5	G	12.5
An_33246658	6.76	G	82.5	A	17.5
An_36897005	6.37	C	82.5	T	17.5

Note: ^a^ The nucleotide shown represents the corresponding nucleotide at the reference genome of Darmor-bzh. ^b^ Nucleotide indicates a nucleotide that differs from the reference genome.

**Table 5:** Candidate gene associated with drought tolerance detected by D value.

Chromosome	Gene ID	Annotation	Gene Name
A01	BnaA01g29390D	9-cis-epoxycarotenoid dioxygenase nced3	*NCED3*
A03	BnaA03g48580D	ABC transporter G family member 9-like	*ABCG*
A03	BnaA03g48550D	Indole-3-acetic acid-amido synthetase	*GH3.5*
A03	BnaA03g48570D	NAC domain-containing protein 72	*NAC72*
A05	BnaA05g22900D	E3 ubiquitin-protein ligase	*UPL6*
A05	BnaA05g22950D	Phytochrome b	*PHYB*
A07	BnaA07g26770D	Glycerate dehydrogenase HPR	*HPR1*
A07	BnaA07g26740D	Heat stress transcription factor a-8	*HSFA8*
A07	BnaA07g26830D	IAA-alanine tolerance protein 1-like	*IAR1*
A07	BnaA07g26870D	Probable WRKY transcription factor 9	*WRKY9*
A07	BnaA07g26850D	Protein indeterminate-domain 14-like	*IDD14*
A07	BnaA07g26910D	Putative two-component response regulator-like APRR6	*APRR6*
A07	BnaA07g26980D	Transcription factor MYB62	*MYB62*
C06	BnaC06g28840D	Photosystem II core complex proteins PSBY	*PSBY*
C06	BnaC06g28780D	Two-component response regulator ARR11	*ARR11*
C06	BnaC06g28950D	E3 ubiquitin-protein ligase RGLG5	*RGLG5*

## Data Availability

All data have been published in the online version.

## Supplementary Materials

The following supporting information can be downloaded at https://www.mdpi.com/article/10.3390/plants13233296/s1, Figure S1: Manhattan plots of GWAS for the Drought Index (DI) value of four traits. Notable peaks above the threshold (−log_10_P = 5.75) are highlighted with arrows; Table S1: Association panel information and trait measurement under control and stress condition; Table S2: Evaluation of drought tolerance based on the D value; Table S3: Clustering of the association panel based on D value; Table S4: Significant SNPs detected by GWAS using D value; Table S5: Candidate genes identified within the 150 kb region surrounding peak SNPs detected by GWAS for D value; Table S6: Regulatory network of drought-responsive transcription factors and their downstream targets; Table S7: Primers used for qRT-PCR.
