# Supplementary material for: Comprehensive Morphological and Molecular Insights into Drought Tolerance Variation at Germination Stage in Brassica napus Accessions
Source: Plants (Basel). 2024 Nov 23;13(23):3296. doi: 10.3390/plants13233296 (PMC11644293; doi:10.3390/plants13233296)

Manhattan plot of DI for germination percentage (GP)

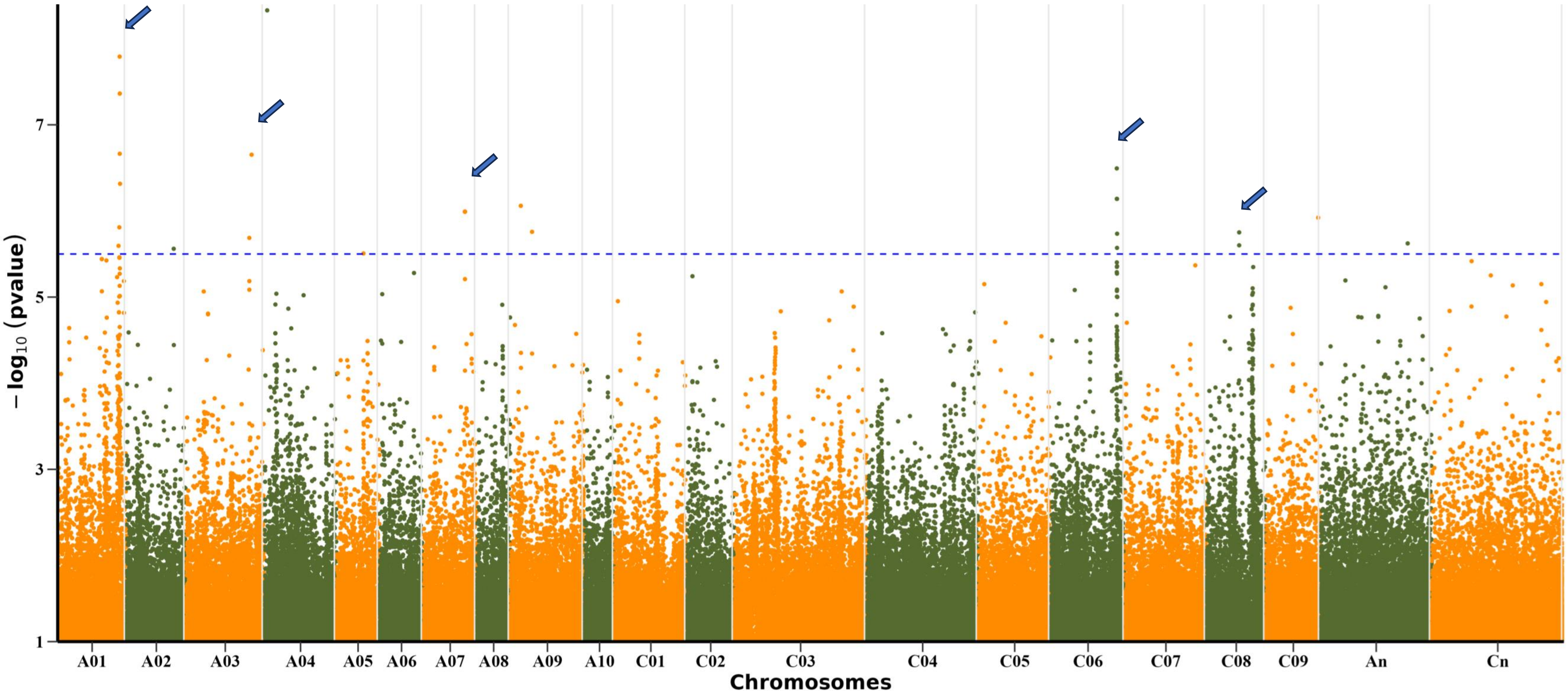

Q-Q plot of GWAS p-value for DI-GP

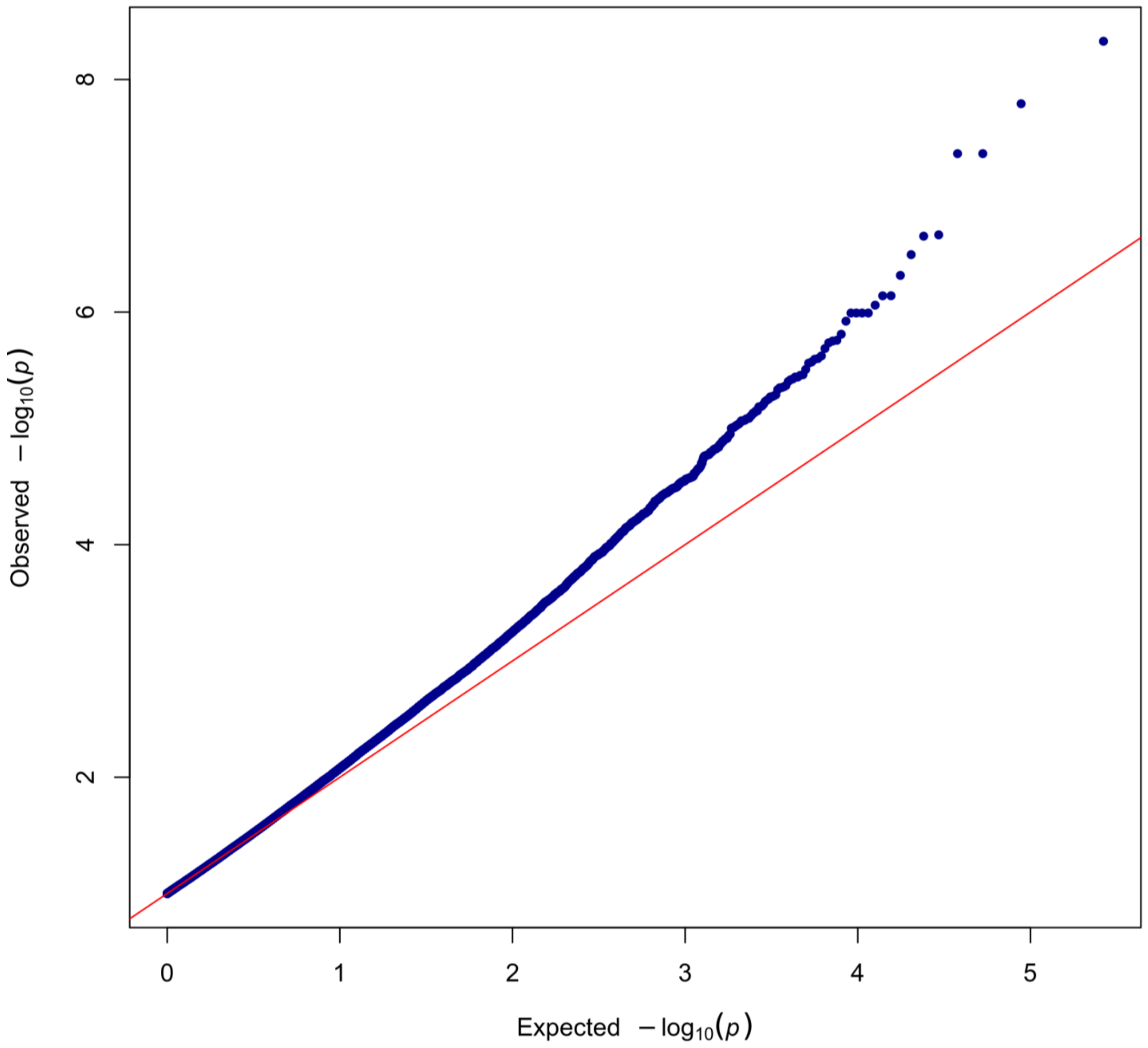

Manhattan plot of DI for fresh weight (FW)

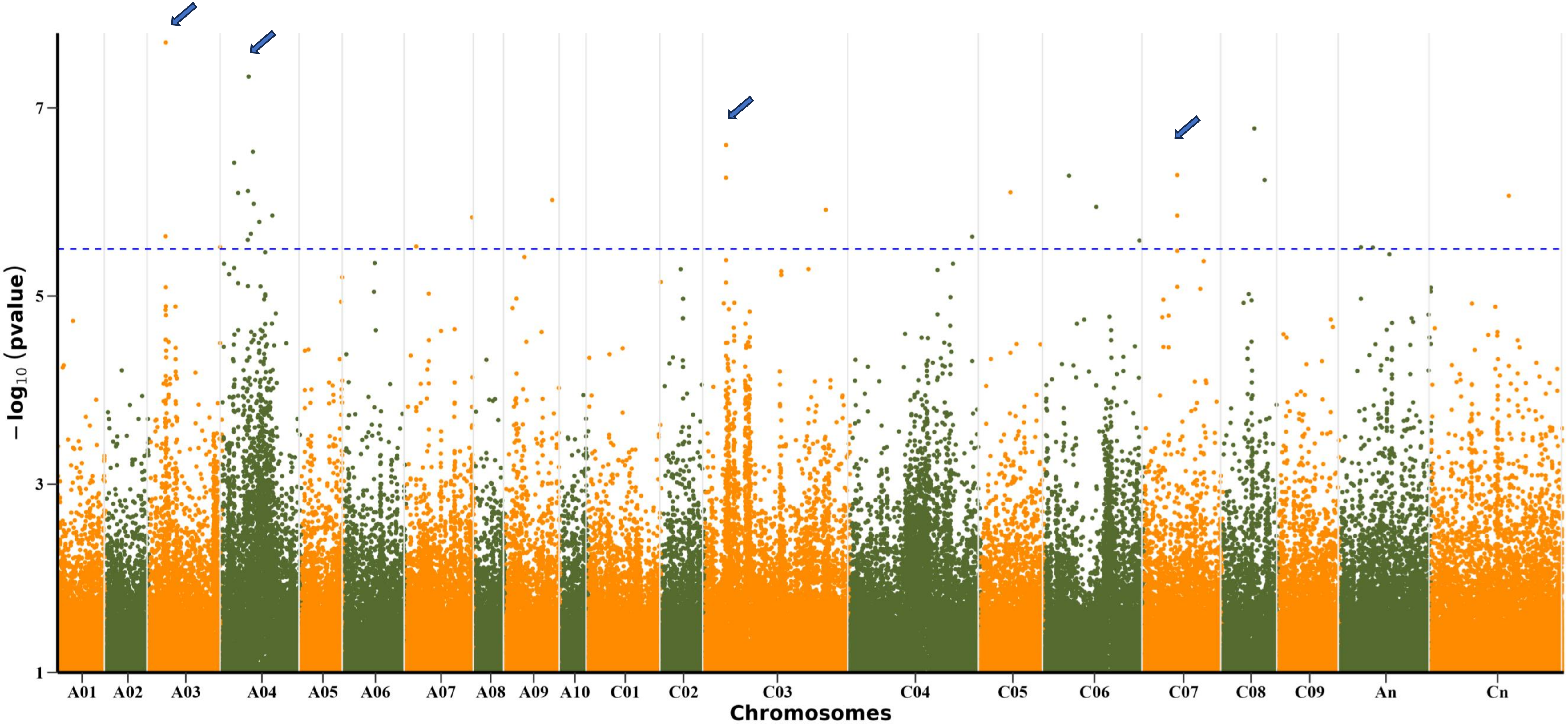

Q-Q plot of GWAS p-value for DI-FW

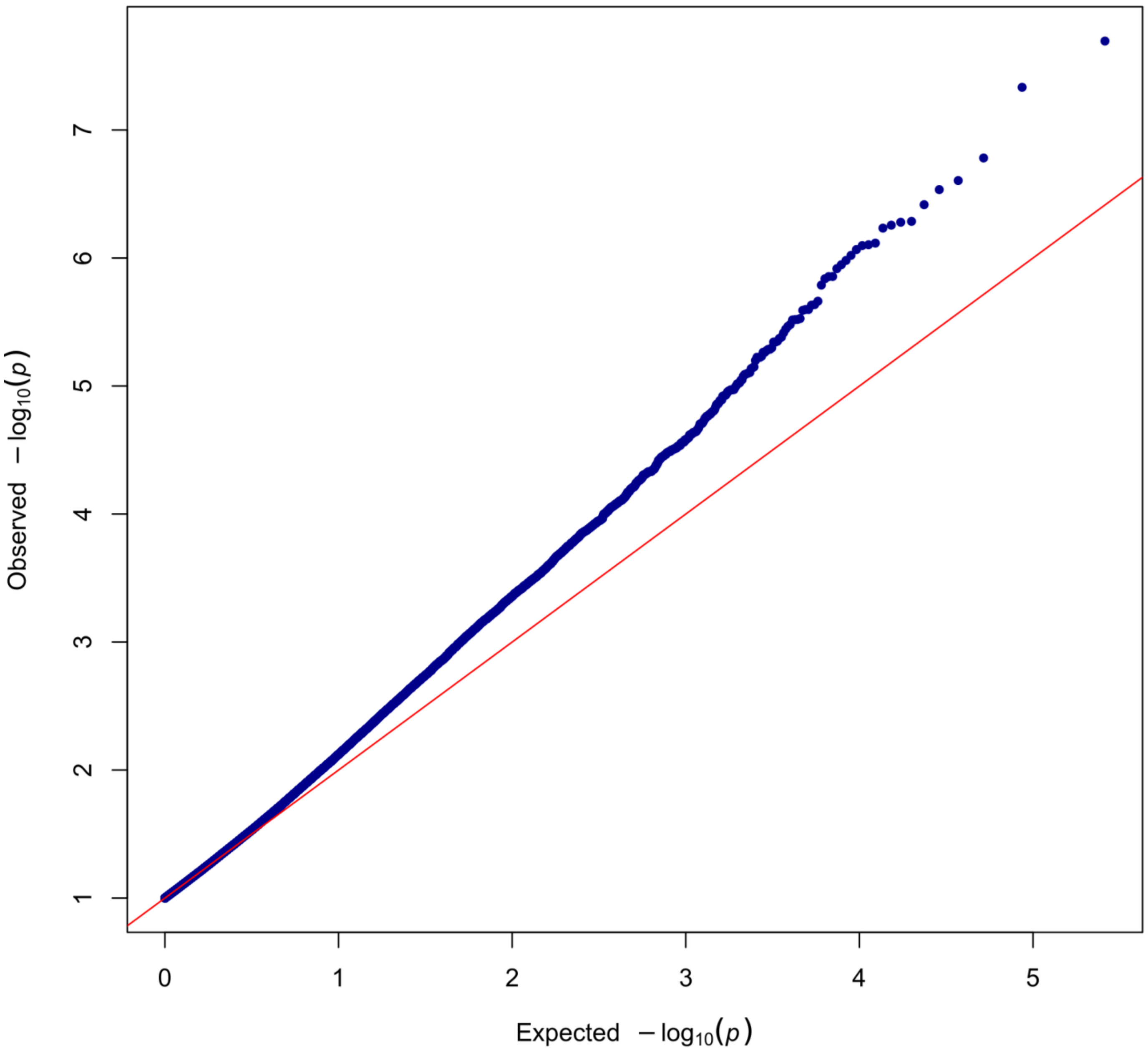

Manhattan plot of DI for shoot length (SL)

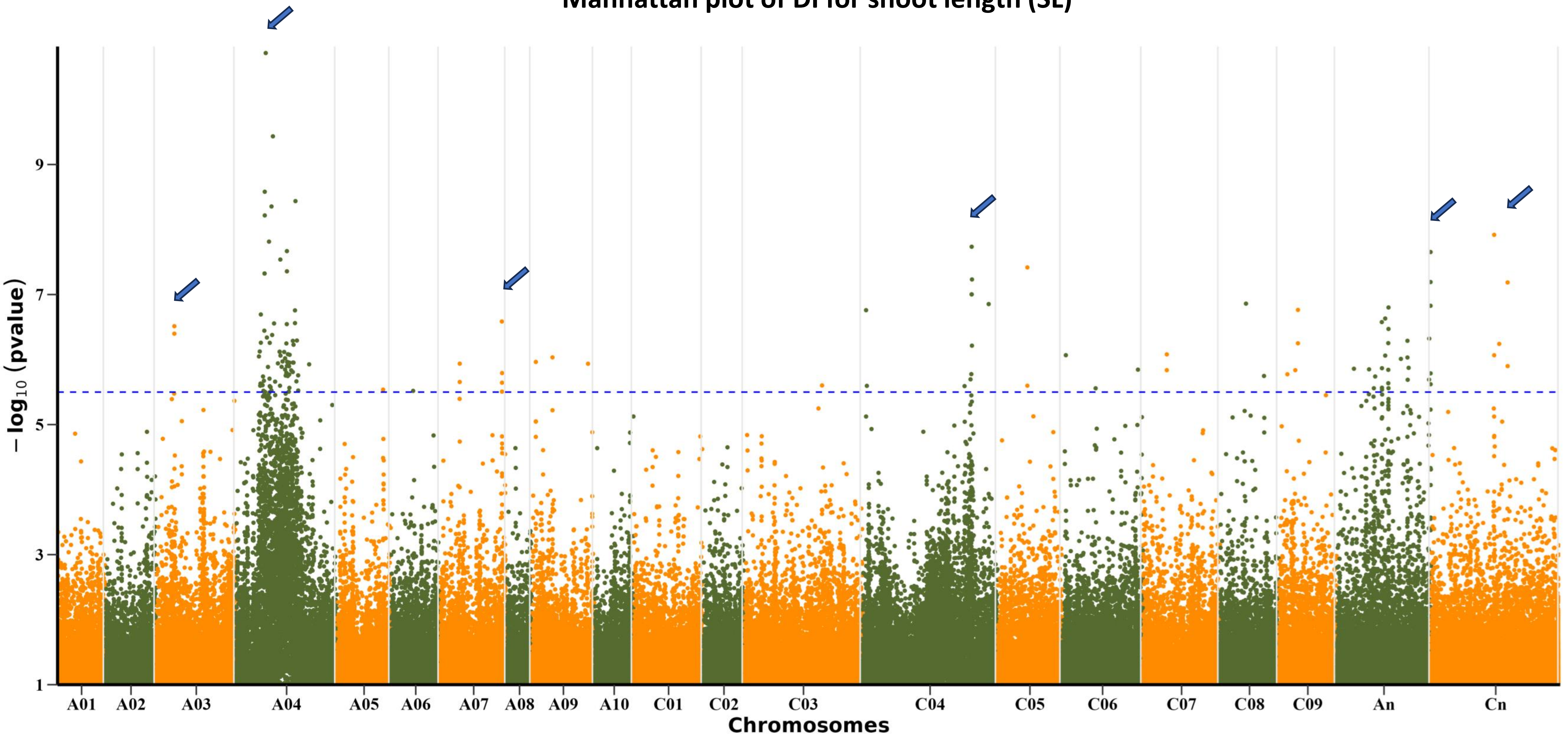

Q-Q plot of GWAS p-value for DI-SL

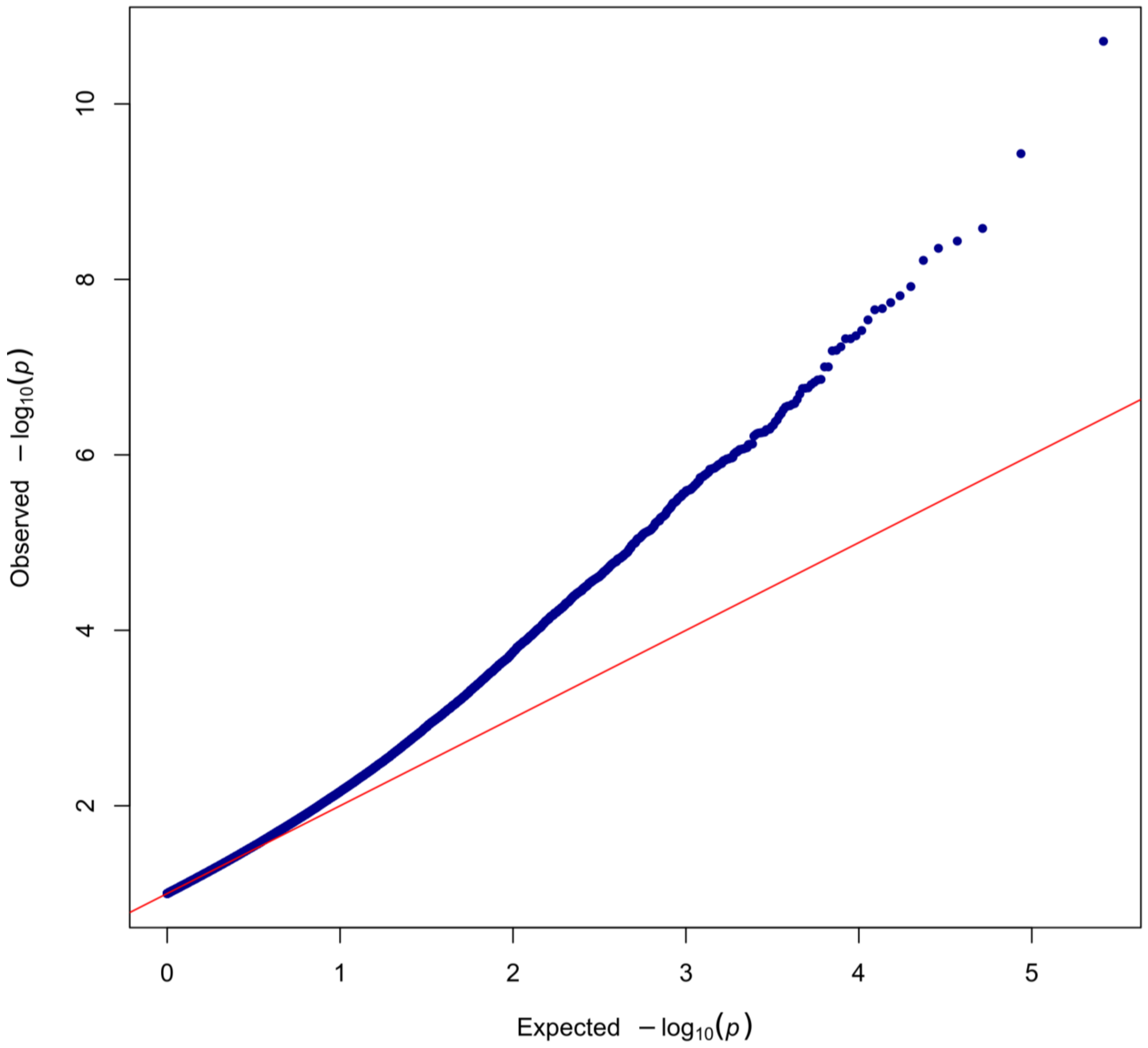

Manhattan plot of DI for root length (RL)

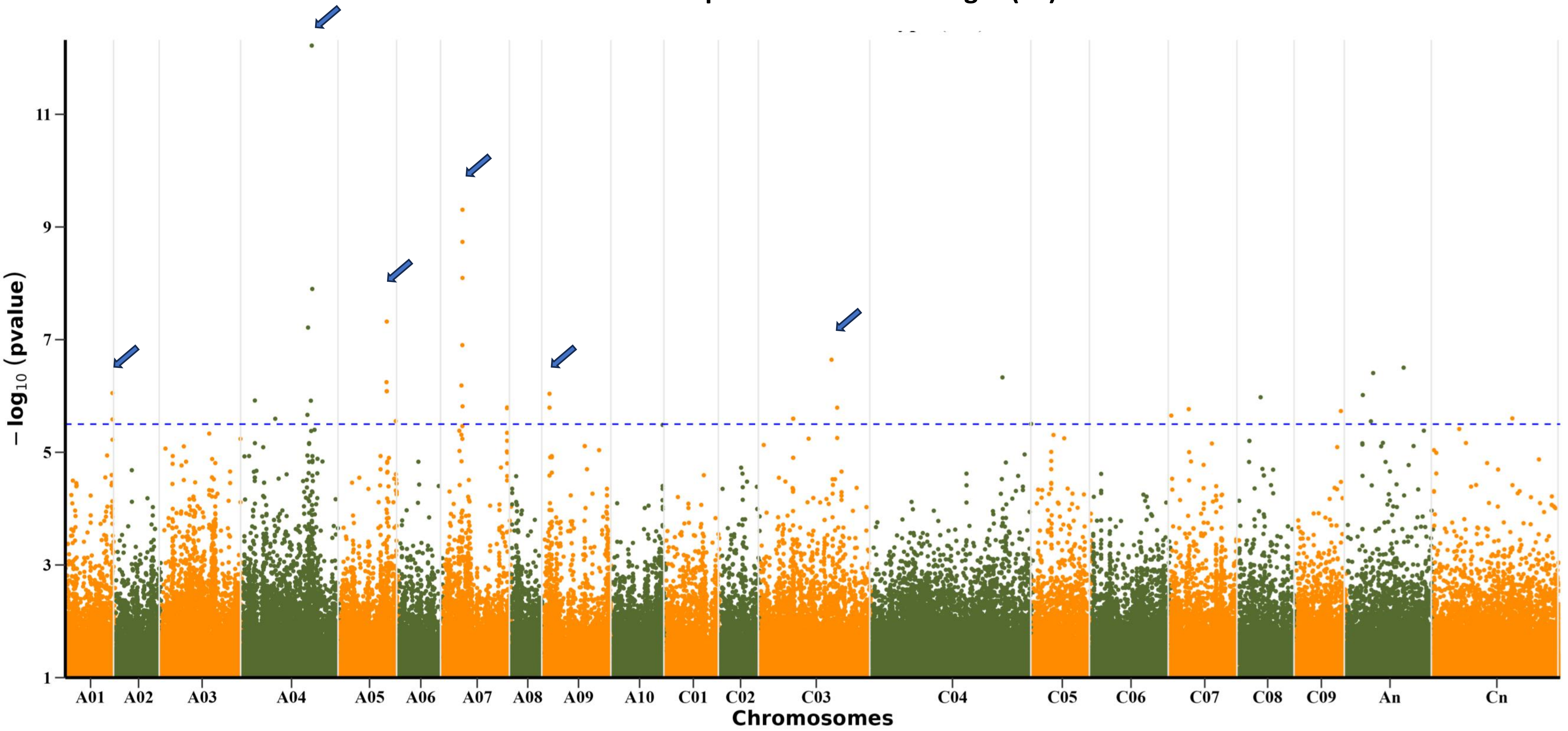

Q-Q plot of GWAS p-value for DI-RL

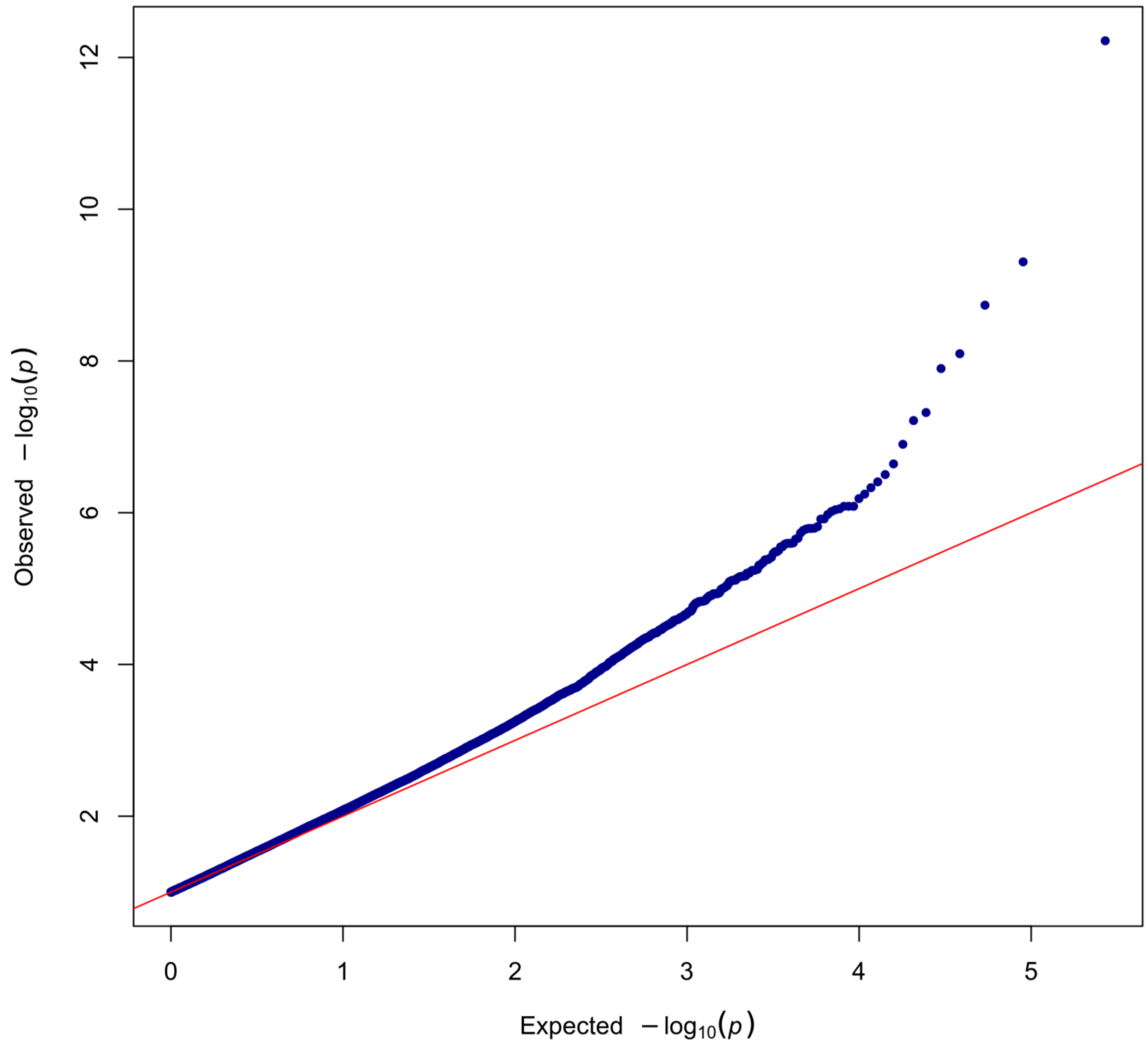

Supplement: Supplementary file 1 [file plants-13-03296-s001.zip › Supplementary Fig. S1.pdf]
